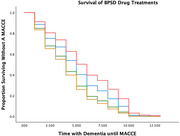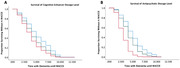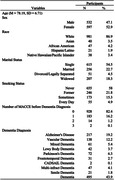# The impact of dementia medications on medical outcomes in older adults

**DOI:** 10.1002/alz.094601

**Published:** 2025-01-09

**Authors:** Haylie M. DeMercy, Colleen A. Brenner

**Affiliations:** ^1^ Loma Linda University, Loma Linda, CA USA

## Abstract

**Background:**

Dementia often includes behavioral and psychological symptoms such as behavioral excitement, mood disorders and psychosis. Antipsychotic drugs are often prescribed alone or in conjunction with cognitive enhancers, however, these drugs can increase risk for adverse cardiovascular/cerebrovascular events (MACCE) and mortality. This study investigated whether those prescribed antipsychotics and/or cognitive enhancers experienced a MACCE more quickly and if medication dosage impacts survival time.

**Methods:**

Data from 1,126 patients aged ≥ 50 or older, with a diagnosis of dementia and behavioral symptoms were studied. We compared four different models of BPSD treatment using Cox Regression Analyses.

**Results:**

Results indicated that there was a significant effect of type of medication on duration until MACCE, (p < .001). The odds of experiencing a MACCE were 96.3% higher for individuals treated with both antipsychotics and cognitive enhancers (p < .001), 78.7% higher for those treated with only antipsychotics (p < .001), and 31.9% higher for those treated with only cognitive enhancers (p = .003). There was also a significant effect of dosage on duration until MACCE, p < .001. The odds of experiencing a MACCE sooner were 238% higher for those on high doses of antipsychotics (p < .001), and 48% higher for individuals on a medium dose (p < .001) compared to low doses. There was also a significant effect of cognitive enhancer dosage on duration until a MACCE, (p < .001). The odds of experiencing a MACCE were 76% higher for individuals on high doses of cognitive enhancers (p < .01) and 38% higher for those on a medium dose (p < .05) compared to low doses.

**Conclusion:**

There is a clear increased risk of adverse medical outcomes when using antipsychotic and cognitive enhancing drugs in the treatment of dementia with behavioral symptoms. The use of antipsychotic medication, particularly at high doses, was associated with the greatest risk of an adverse medical outcome. Use of antipsychotic medications in this population should include close monitoring for cardiovascular/cerebrovascular events.